# Floral Nectar Chemistry in Orchids: A Short Review and Meta-Analysis

**DOI:** 10.3390/plants10112315

**Published:** 2021-10-27

**Authors:** Emilia Brzosko, Paweł Mirski

**Affiliations:** Faculty of Biology, University of Bialystok, Ciołkowskiego 1J, 15-245 Bialystok, Poland

**Keywords:** life form, nectar concentration, nectar presentation, Orchidaceae, plant–pollinator interactions, sugars, sugar ratio

## Abstract

Nectar is one of the most important flower traits, shaping plant–pollinator interactions and reproductive success. Despite Orchidaceae including numerous nectariferous species, nectar chemistry in this family has been infrequently studied. Therefore, the aim of this study is to compile data about nectar attributes in different orchid species. The scarcity of data restricted analyses to sugar concentration and composition. Our results suggest that the most important factor shaping nectar traits in orchids is the pollinator type, although we also found differentiation of nectar traits according to geographical regions. In spurred orchids, the length of the spur impacted nectar traits. We recommend the development of studies on nectar chemistry in orchids, including a wider range of species (both in taxonomic and geographical contexts), as well as extending the analyses to other nectar components (such as amino acids and secondary metabolites). The nectar biome would be also worth investigating, since it could affect the chemical composition of nectar. This will enrich the understanding of the mechanisms of plants–pollinators interactions.

## 1. Introduction

As one of the largest families among flowering plants, Orchidaceae is characterized by an unusual diversity of flowers (regarding, for instance, flower structure, color, and odor), which is attributed to natural selection on functional traits associated with the behavior, physiology, and anatomy of pollinators. This diversity is reflected in the differentiation in pollination mechanisms and reproductive strategies. The common strategy among orchids is floral deception. Most authors have reported that about 30–40% of orchids deceive pollinators through sexual or food deception ([1,2,3,4,5] and literature cited therein). The other representatives of Orchidaceae reward pollinators by producing different attractants, such as nectar, fragrance, oils, resin, and wax [6]. The rewarding species achieve higher reproductive success than the deceptive ones, and among the rewards offered by orchids, nectar is the most effective [3,7,8]. The role of nectar presence for pollination effectiveness, including orchids, is well documented [3,8,9,10,11,12,13]. Its importance for reproductive success has been confirmed by experiments with manipulations of nectar or nectar spurs, which proved that nectar presence increases the frequency of pollinators’ visits and their efficiency [7,14,15,16], and may even change pollination modes [17]. Less attention has been paid to the impact of nectar quantity and quality on reproduction.

Nectar quantity and quality vary widely in different species or whole taxonomic groups, and are differentiated in space (between populations or individuals in a given population) and time (between years and depending on flower development phase) [9,10,11,12,18,19,20,21,22,23,24,25]. Despite the variability in nectar properties, some patterns appear. In most cases, three main sugars are present in flower nectar (sucrose, glucose, and fructose), and rarely with the addition of other carbohydrates (e.g., mannose, xylose, and maltose) [13,20,21,23,26]. Despite floral nectar being constituted from the same main sugars, the concentration and ratio between sugars vary substantially, although nectar is most often sucrose dominant [13,20,21,26]. The predominance of hexoses is less common [12,22,27,28,29]. The sugar amounts and proportion depend on various pollinator types [21,30,31], mainly on the structure of their mouth apparatus, which is adapted to use nectar with an adequate sugar concentration [30,31,32]. The preferences of pollinators to different sugar concentrations and proportions are also a result of their energetic needs due to the different costs connected with their body size and behavior. Large pollinators require more energy than smaller ones, thus are dependent on plants that produce larger amounts of more concentrated nectar [33,34]. Heil [35] suggested some physiological determinants of pollinators’ preferences: while most hummingbirds and ants prefer sucrose-rich nectar, some nectarivorous birds and ants prefer sucrose-free nectar because they lack invertase and are not able to assimilate sucrose. The preferences of pollinators are also related to another important group of nectar components, amino acids (AAs). Although the amount of AAs in floral nectar is significantly lower than that in sugars, they constitute an important part of the diet of animals feeding on nectar. Apart from their nutritional function, AAs influence the taste of nectar, and this secondary role is even more important than the nutritive value according to some authors [36,37]. Similar to sugars, the composition of AAs in nectar is related to particular pollinator types. For example, plants pollinated by birds or flies are characterized by a lower concentration of AAs, whereas those pollinated by butterflies produce nectars with higher AA concentrations [36].

Nectar in orchids, as in other plants, is secreted and accumulated in different parts of the flower. In the Orchidaceae, it may be located in shallow and cup-like structures, at the base of the labellum, in long spurs, at the base of the flower alongside the ovary and on the side lobes, or along the central groove of the labellum [38]. The nectar exposition and, thus, its accessibility significantly determines plant–pollinator interactions and, consequently, the effectiveness of pollination. Nectar located inside the corolla or in the spur is protected against evaporation and is available for specific, restricted groups of pollinators, whereas exposed nectar is more vulnerable to evaporation and robbery, but is available for a wide range of pollinators, which often differ with respect to body size, mouth apparatus, and requirements [21,26,39]. Additionally, nectar accumulated in concealed nectaries is usually dominated by sucrose, whereas in open flowers, by glucose and fructose [21,40,41,42].

Despite nectariferous orchids being numerous and the role of floral nectar for their fitness being indisputable, the chemical composition of their nectar has been infrequently studied. The available data concern only a small number of species and are most often fragmentary. Some of the earliest information was provided by Percival [40], who included eight orchid species in her review on the sugar proportions in almost 1000 species, and by Jeffrey et al. [43], who reported the sugar content in orchid species and their hybrids. Unfortunately, the latter only provided data on the presence of particular sugar types and did not distinguish between floral and extrafloral exudates.

To date, no review has been published on nectar chemistry in Orchidaceae. Therefore, the aim of this study is to bridge this gap. Considering the available knowledge on nectar properties in plants and as the Orchidaceae are an example of a family that is known as specialists with respect to pollinators, we assumed that nectar traits should be dedicated to certain pollinators or their groups. Thus, nectar chemistry should differ between orchids pollinated by distinct pollinator types. We also tested the geographical patterns of nectar properties for the influence of life form and for the method of nectar presentation on nectar characteristics.

## 2. Results

### 2.1. Description of the Database

The database included 116 records that reported nectar in orchids, among which 106 concerned sugar concentration (Table S1). In some papers, we found data about the amounts of particular sugars or their percentages, which allowed us to calculate sugar ratios in 43 cases. Altogether, the data included in this meta-analysis were obtained from 64 articles published between 1984 and March 2021. The dataset included 110 orchid species belonging to 36 genera. For two species (Satyrium halackii and S. cristatum), subspecies or variants were also studied (Table S1). Three species (Habenaria johannensis, Mystacidium pusillum, and Platanthera chlorantha) were studied in two different places. Some genera in the dataset were represented by numerous species, with the richest being Habenaria (17 species), Satyrium (15 species), Mystacidium (8 species), and Aeranthes (7 species) (Table S1). The majority of data were derived from Africa (57) and South America (32); from Europe, Asia, Australia, and North America, we noted 9, 9, 6, and 3 records, respectively. Among the species analyzed, 66 were terrestrial, 38 epiphytic, and 2 epilithic. Most species (99) were specialists according to pollinators, and 7 were generalists. Specialist taxa were pollinated most often by moths (49), and the remaining by birds (19), bees (20), butterflies (9), flies (5), wasps (5), and beetles (1). Nectar was accumulated in spurs and open nectaries in 87 and 23 of the studied species, respectively.

### 2.2. Overall Characteristics of Nectar in Orchids

**Figure 1:**
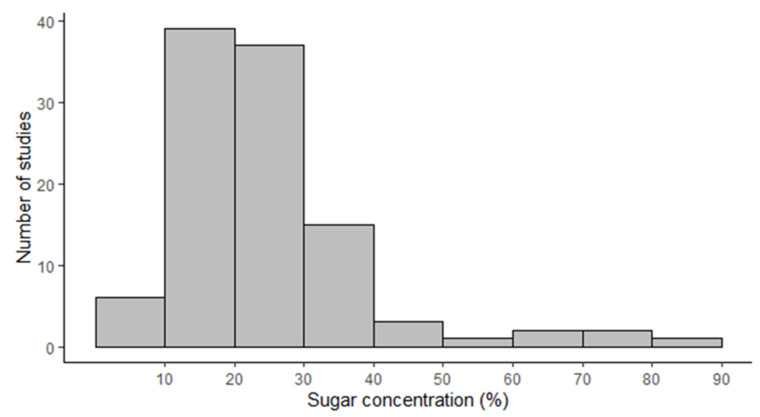
Histogram of sugar concentration measured in 106 different orchid species around the globe.

The mean sugar concentration in the nectar of all orchid species was 24.29% ± 13.94%, ranging from about 3.5% (Neottia ovata and Luisia teres) to 71% and 90% in two species from the Acrolophia genus (A. cochlearis and A. micrantha) (Table S1). In 49 cases (46.23%), nectar concentration was ≤ 20%, and in 98 cases, it was 92.5%. For 106 of the analyzed species, the concentration was ≤ 40% (Figure 1). Among 43 species for which data on sugar ratios were available, 33 (80.5%) secreted sucrose-dominant nectar and two others secreted sucrose-rich nectar (Table S2). Only in one species (Platanthera chlorantha) nectar was dominated by hexoses; in six species, including four generalists, the nectar was hexose rich.

The most supported model (Table 1) explaining nectar concentration (AIC = 123.3) in orchids involved 4 variables: continent, climatic zone, pollinator type, and spur category, which explained over 40% of the variance. This model outperformed the next model, which had the second-lowest AIC value by ΔAIC = 3.5 (Table S3).

### 2.3. Geographical Patterns

**Figure 2:**
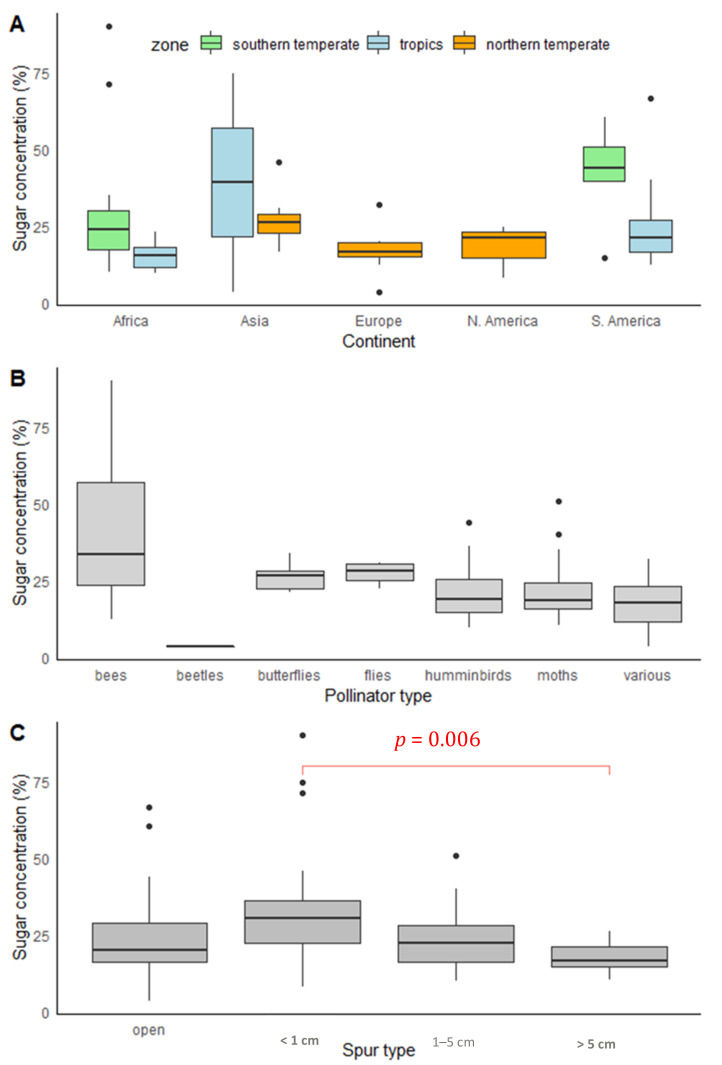
Sugar concentration in flowers of 106 orchid species around the globe depending on their geographical location (**A**), pollinator type (**B**), and spur type (**C**). Significant differences in Tukey’s post hoc test between spur length categories is indicated by a red line and red font.

The linear model showed that orchids in Asia and, to a lesser extent, in South America had higher concentrations of sugar in their nectar. Higher sugar concentration was also a feature of orchids in southern temperate zones (Table 1, Figure 2A). The most concentrated nectar was noted for Asian orchids (29.94% ± 20.15%) and the lowest for species from Europe and North America (16.89% ± 8.04% and 17.90% ± 8.77%, respectively). In the most numerous orchid groups from Africa and South America, the mean values of this parameter reached 23.23% ± 13.31% and 27.18% ± 13.96%, respectively. The nectar concentration of orchids from Africa was 2 times higher than that of those on the islands in the Indian Ocean (Madagascar, Reunion, and Mauritius) (25.0% ± 13.54% vs. 12.60% ± 2.89%, respectively). The sucrose/hexose ratio (R1) differed significantly only in South America (Table S4), being higher in tropical than in temperate zones (*p* = 0.03). The fructose/glucose ratio (R2) differed significantly between continents (Table S4; χ^2^ = 8.34, *p* = 0.04). 

### 2.4. Pollinators

Orchid species pollinated by different pollinator types produced nectar with different sugar concentrations (Figure 2B). The highest concentration of sugars in the nectar was noted for bee-pollinated orchids (40.21% ± 22.75%). The concentration for bird- and moth-pollinated species was two times lower (Figure 2B). Butterfly- and fly-pollinated orchids had nectar with about 26% and 27% sugars, respectively. The most diluted nectar was found to be produced by generalists (17.82% ± 10.17%). In the single species (*Luisia teres*) pollinated by beetles, the sugar concentration was only 3.5%. The variance of sugar concentration differed between pollinator types (Bartlett’s K2 = 24.508, *p* = 0.0002), showing a relatively moderate range for most of the pollinator groups, but high in the case of orchids pollinated by bees (Figure 2B).

Sucrose/hexose and fructose/glucose ratios (R1 and R2, respectively) did not depend on pollinator type (Table S2), although these values fell within a wide range. For example, R1 values ranged from 1.22 ± 1.20 in generalists to 51.50 ± 68.59 in orchids pollinated by flies. Statistical tests showed no significant differences either in sugar concentration (Table S2) or in sugar ratios (Table S4) between specialists and generalists, possibly due to the small amount of data for generalists. Nevertheless, the average R1 for specialists vs. generalists was 17.42 vs. 0.73, but 1.20 vs. 0.88 for R2.

### 2.5. Sugar Concentration According to the Life Form of the Orchids

The nectar of terrestrial orchids contained more sugar than of epiphytic and epilithic ones (25.96 ± 13.80% vs. 21.52 ± 13.90%, respectively), although differences were not statistically significant. Neither habitat nor life form were included in the most supported model explaining sugar concentration (Table S1). Similarly, we noted no statistically significant differences between orchids representing particular life forms (epiphitic vs. terrestrial) or their sugar ratios (R1: U = 156, *p* = 0.11; R2: U = 119, *p* = 0.28).

### 2.6. Nectar Presentation

Sugar concentration did not differ between species with open nectaries (25.19 ± 17.43%) and those with nectar deposited in spurs (24.10 ± 13.23%). The R1 ratios of nectar located in spurs and in open nectaries were similar (17.6 vs. 13.1, respectively). Among orchids with open nectaries, five species produced nectar dominated by hexoses (Epipactis atropurpurea, Eulophia alta, Maxillaria anceps Stenorrhynchos orchioides, and Neottia ovata), seven species by sucrose (Beadlea dufrae, Caladenia arenaria, C. colorata, C. versicolor, C. paludosa, Elleanthus brasiliensis, and Pelexia bonariensis), and, in one species (Caladenia nobilis), the sucrose/hexose ratio was equal to 1.

The spur length was differentiated among pollinator types. The longest spurs (and simultaneously, the most differentiated) characterized orchids pollinated by moths (68.41 ± 72.19 mm) and the shortest characterized those pollinated by bees (9.74 ± 6.80 mm). The spurs of orchids pollinated by other pollinator types were more than two times shorter than those of moth-pollinated species (Figure S2).

**Figure 3:**
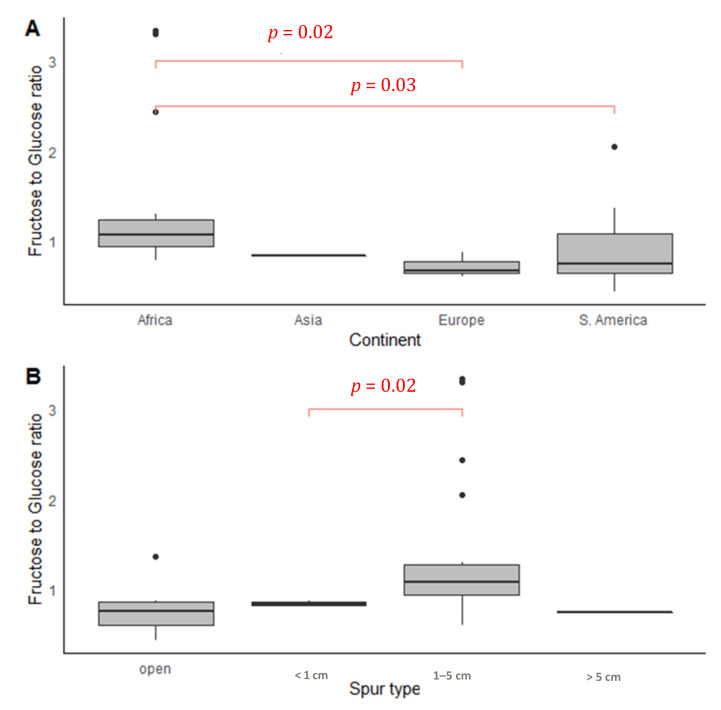
Fructose-to-glucose ratio in the nectar of 30 orchid species compared between continents (**A**) and spur type (**B**). Significant differences in Dunn’s multiple comparison test are indicated using red lines and red font.

The sucrose/hexose ratio between species with short and medium spur lengths differed significantly, with the latter reaching the highest ratio of those sugars (Figure 3B, *p* = 0.05). The fructose/glucose ratio (R2) was close to significant between spur types (Table S4). The spur length influenced pollinator numbers (r = −0.28, *p* < 0.05).

### 2.7. Nectar as Taxonomic Trait

Species included in this meta-analysis, belong to only two subfamilies, Epidendroidae and Orchidioidae. All nectar traits appeared at similar levels in these two subfamilies and their variance showed no significant differences in sugar concentration (Z = −0.57, *p* = 0.57), R1 (Z = −0.60, *p* = 0.55), or R2 ratio (Z = 0.61, *p* = 0.54), or in the share of sucrose (Z= −0.19, *p* = 0.85). The variance in sugar concentration did not differ between the different genera of Orchidaceae family (K2 = 9.57, df = 11, *p* = 0.57), although some genera were located on different edges of the overall variance (Figure S1). The variance in sugar concentration, compared between the genera with most nectar records, Habenaria and Satyrium, was also non-significant (Z = 0.31, *p* = 0.75). The lowest sugar concentration was observed for Angraecum genus, and the highest for Rodriguezia and Brownleea. Comparing all analyzed genera, the sucrose:hexose ratio (R1) showed no significant differentiation (χ^2^ = 8.54, df = 6, p = 0.201), whereas fructose:glucose ratio (R2) was close to significantly differentiated (χ^2^ = 12.23, df = 6, *p* = 0.057). Only the share of sucrose showed significant differences (χ^2^ = 11.19, df = 5, *p* = 0.048).

## 3. Discussion

Our survey confirms the paucity of information about the composition of nectar in orchids. We found data on nectar for 110 orchid species belonging to 36 genera, covering less than 0.5% of all known representatives of the Orchidaceae family, with a size of around 25,000 species. This is startling, because this family has received considerable attention due to its functional diversity, and since most of orchids secrete floral nectar. Our analyses show that nectar is as variable as the other properties of Orchidaceae. At the family level, its concentration fell within a wide range, from 3.39% to 90%, but in 92.08% of cases, it was ≤40%. This is within the range reported for other plants [13,21,26,30,39], but, to the best of our knowledge, it is one of the widest ranges amongst the known angiosperm taxa. The mean nectar concentration for Orchidaceae is low (24.29% ± 13.94%) in comparison with other families [44], but is similar to Bromeliaceae, which are pollinated by a comparable pollinator group [45]. This may be related to the large proportion of bird- and moth-pollinated species in the whole data set, which are characterized by the lowest nectar concentration. The majority of orchid species (80.5%) secrete sucrose-dominant nectar. Percival [40] noted that among eight orchid species analyzed, five were characterized by sucrose-dominant nectar and three by hexoses. The dominance of sucrose has been observed in other taxonomic groups, e.g., Bromeliaceae [45], Caryophyllaceae [46], and Gentianales [47]. Petanidou [20] found different sugar ratios in distinct families in phryganic vegetation.

### 3.1. Spatial Variation in Nectar Traits

Our results showed that nectar properties vary in space at different levels of biological organization. Higher nectar concentration was a feature of orchids in southern temperate zones and the lowest concentration was found in species growing in the northern temperate zone (Europe and North America). Despite these continents being represented by a relatively low number of species, the concentration of nectar in both groups occurred at the same level. The nectar concentrations of the most numerous orchid groups in our analyses (from Africa and South America) were also similar (23.23% ± 13.31% and 27.18% ± 13.96%, respectively). The most concentrated nectar was noted for Asian orchids, which may be explained by the contribution of two orchid species with the highest concentration in the entire data set (90% and 71%). The interesting finding of our analyses is that the nectar concentration of orchids from continental Africa was 2 times higher than that of orchids from Indian Ocean islands (Madagascar, Reunion, and Mauritius) (25.0% ± 13.54% vs. 12.60% ± 2.89%, respectively). This difference may be caused by the distinct relationships between plants and pollinators on mainland and islands, which might have resulted from different periods in orchid evolution in these two areas. This explanation was inspired by results of Claessens et al. [48], who documented the differences in spur length of *Habenaria tridactylites* with island age, which decreases from the oldest to the youngest islands. We also found geographical differentiation in the proportions of particular sugars. In South America, the sucrose/hexose ratio was higher in tropical than in temperate zones. The fructose/glucose ratio differed significantly between continents. Differences in nectar properties are partially connected with the differing climatic conditions around the globe. High-hexose nectars require more water [34]. For example, Petanidou [20] suggested that the predominance of sucrose in species from the Mediterranean region is due to the warm, dry climate. The few studies documenting nectar properties between continents or climatic zones preclude deep comparison in this respect. Chalcoff et al. [49], reviewing data on more than 1000 species of angiosperms, found a higher sucrose proportion for temperate compared with tropical and subtropical species. Galetto and Bernardello [50] and Chalcoff et al. [51] noted differences in the nectar composition of plants from distinct biogeographical regions in Argentina.

Variability in nectar chemistry, as in orchids, is noted at the lower spatial scales: between different populations of the same species, within populations, or even between flowers of individual plants [26]. Inter-population differentiation is well documented, for example, in studies on *Gymnadenia conopsea* [9], two species of *Platanthera* [12], and *Neottia ovata* [29], in which nectar concentration, amount of sugars, and AAs sometimes differ among populations. A paper included in our review reported within-species variation in nectar concentration (the largest differences were documented by Nilsson and Rabakonandrianina [52], Rodríguez-Robles et al. [53], Neubig et al. [54], and Pansarin and Ferreira [55]). Intraspecific variation in nectar traits is explained through differentiation of abiotic factors, mainly the properties of habitats, e.g., soil chemistry [9,22,29,36]. In the case of epiphytic orchids, nectar variability may reflect the different photosynthetic activities of the plants growing in different parts of trees with distinct exposure to light and, consequently, to temperature and water availability. Brzosko and Bajguz [12] found differences in some nectar traits between meadow and forest populations of *Platanthera bifolia* and *P. chlorantha*. Wasserthal [18] found lower nectar concentration in *Angraecum sororium* growing under a tree canopy. The importance of light as a factor influencing nectar properties was also noted by Nocentini et al. [22].

Finally, the spatial variability in nectar properties may occur due to pollinators’ shifts within the geographical range of a given species [18,50,51]. This fact may impact many different flower traits, as pollinators are a highly important selective pressure, driving evolutionary changes, including the nectar properties, of the plants they visit.

### 3.2. What Kinds of Nectar Do Orchid Pollinators Prefer? 

One of the most common and repeated opinions in papers on nectar studies is that nectar traits are linked to pollinator types [21,32,39], although some results are not in accordance with this pattern [47,51,52]. The results of the former authors support the idea that sugar composition responds to pollinator-mediated selection, because plants produce nectar in order to attract pollinators, which is optimized to the energy requirements of pollinators. The results of our analyses generally agree with the predominant model, that orchids pollinated by distinct groups of pollinators differ in nectar sugar concentration. Similar results were obtained for another family with a wide range of pollinators, the Bromeliaceae [45]. Nevertheless, in some orchid groups, for example, Sobralieae, sucrose concentration was not related to pollinator type [54]. In general, the bee-pollinated orchids were characterized by the most concentrated nectar (40.21% ± 22.75%). In addition, the highest values of nectar concentration were noted in this orchid group (90% and 71%). Similar values (~ 41%) of sugar concentration in the nectars of bee-pollinated species, irrespective of continent and community type, were observed by Pamminger et al. [56] in their meta-analysis of various plant families. In tropical plants, Pyke and Waser [57] found a slightly lower nectar concentration in flowers pollinated by bees (~ 35%), whereas Wolff [47] noted a less concentrated nectar (25.9%) in melittophilous species from Gentianales growing in Ecuador. Bee-pollinated Gesneriaceae produce nectar of a relatively low concentration (28.7%, [58]). The above-mentioned data suggest that bees usually prefer more concentrated, and thus higher-viscosity, nectar, compared with other pollinators, which is connected to the mode of nectar intake and the energetic needs of these insects [59]. Notably, the range of values of nectar concentration in bee-pollinated orchids is considerably wider than in species pollinated by other pollinators, as reported by Pamminger et al. [56]. Based on experimental and field studies (literature cited in [56]), they concluded that a sugar concentration of 65–35% is optimal, 35–20% is adequate, and <20% is low quality for bees. Given this information, bee-pollinated orchids offer nectar suitable for this group of pollinators.

The nectar of orchids pollinated by birds and moths was two times less concentrated (~20%) than the nectar of those pollinated by bees. This agrees with the statement that moths, as sucking feeders, prefer less concentrated nectar [59,60], and Josens and Farina [61] found that hawk moths achieve peak intake at a 34% sucrose concentration. Similar or lower sugar concentrations in nectar for bird- and moth-pollinated plants have been reported by other authors [45,47,57,62]. In contradiction to our results are those published by Chalcoff et al. [51], who found that species pollinated by nocturnal insects in a temperate forest in South America produce the most concentrated nectar among the studied plants (44.6% ± 5.5%). Simultaneously, these authors documented the lowest nectar concentration for hummingbird-pollinated plants (29.0% ± 3.6%), but this value is higher than that obtained in our study as well as in other studies [39,45,47,58]. Vandelook et al. [39], studying Balsaminaceae, found that bird-pollinated species produce nectar of low concentrations, which, together with their large amounts and extremely high sucrose content, form a combination of unique traits. This shows that uniform selection on nectar traits in bird-pollinated species is strong and drove the evolution of similar nectar properties among a wide range of plant species adapted to bird pollination.

In the intermediate position within the nectar concentration range, observed in Orchidaceae, we noted butterfly- and fly-pollinated orchids (about 26% and 27%, respectively). Butterflies, like moths, belong to the group of sucking feeders, thus prefer less concentrated nectar. Willmer [31] reported that the optimal feeding rates for Lepidoptera occur at sugar concentrations of 35–45%, and stated that concentrations above 30–40% are difficult to suck for most lepidopterans due to their long tongues, which require dilute, non-viscous nectar. The nectar concentration of the next group, fly-pollinated orchids, was the least variable (22.1–30.5%). This result contradicts that of Wolff [47], who found a wide range of sugar concentration (13–59%) and simultaneously a higher nectar concentration (31%) for species from Gentianales exclusively visited by flies. An extremely low concentration (< 5%) in fly-pollinated Balsaminaceae species was observed by Vandelook et al. [39], as well as other authors [34,36,63]. The variability in nectar concentration among fly-pollinated plants can be explained by the wide variation in this insect group. Flies may possess both short and long tongues, thus, independently of the length of mouth apparatus, distinct species groups feed on nectar of different concentrations.

The preferences of pollinators are affected not only by the nectar concentration, but also by the sugar composition. The nectar of orchids is dominated by sucrose regardless of pollinators, excluding generalist species, which is in accordance with the most common pattern [13,21,26,28,34]. The sucrose/hexose ratios were highly variable. In bird-, moth-, and wasp-pollinated orchids, the sucrose/hexose ratios were lower than in orchids pollinated by bees or butterflies. These results contradict those of Chalcoff’s et al. [51], in which hummingbird- and nocturnal-insect-pollinated species from a South American temperate forest showed a higher sucrose proportion than diurnal-insects-pollinated plants. Sucrose-dominant nectar in ornithophilous, as well as in the majority of flowers classified as sphingophilous, was found by Nicolson and Fleming [64], Wolff [47], Chalcoff et al. [51], and Vandelook et al. [39]. Among bird-pollinated species, nectar may be differentiated; in hummingbird-pollinated plants, it is dominated by sucrose, whereas in passerine-pollinated plants, it is dominated by hexoses [50,51]. Nicolson and Fleming [64] found a bimodal pattern in sunbird-visited plants: some species produced high sucrose nectar, whereas nearly half produced nectar with less than 10% of sucrose.

The less concentrated nectar with a lower sucrose proportion in moth-pollinated orchids resulted from a part of the species included in our analyses being pollinated by moths with especially long proboscises, which require especially diluted nectar.

Studies on bee-pollinated plants have documented contrasting results with regard to sugar composition preferences. Baker and Baker [32] found that short-tongued bees prefer hexose-rich nectars, whereas Petanidou [20] noted that the nectars of bee-pollinated Mediterranean species across a number of families are dominated by sucrose. Peter and Johnson [65] showed that bees are a heterogeneous group, with nectar preferences varying from low- and medium-sucrose, and suggested that these contrasting results in sugar composition are not a particularly critical aspect for bee pollination. The next pollinator group, flies, prefer hexose-dominant or hexose-rich nectar according to Baker and Baker [30], Gardener and Gillman [36], Nicolson [63], and Abrahamczyk et al. [34]. This was confirmed by studies on Gentianales [47] and Balsaminaceae [39]. In our studies, fly-pollinated orchids produced sucrose-dominant nectar, although this result should be taken with caution, because it is based on only two species. The wasp-pollinated orchids also produce nectar dominated by sucrose. Similarly, wasps in the Mediterranean phrygana preferred nectars of high sucrose content Petanidou [20].

It is interesting to consider nectar traits in the context of specialization. A specialized pollination mode is associated with a high sucrose content in nectar [32,60,64,66], whereas generalists often prefer hexose-rich nectar, and sucrose-rich nectar can be even toxic for some of them [34]. Hexose-rich nectar, the uptake of which is easier compared with sucrose, may be an adaptation and advantage for attracting a wide range of nonspecialized pollinators. Johnson and Nicolson [66] found a clear distinction between the nectar sucrose content of specialized (40–60%) and generalized (0–5%) bird-pollinated species. Although we did not find statistically significant differences in the sucrose/hexose ratio between specialists and generalists, this ratio reached, on average, about 17 in specialists and 0.73 in generalists. Moreover, in generalists, glucose dominates over fructose. To confirm whether the sugar composition found in our analyses is the rule, more data are needed, especially for generalist orchids, since our data on sucrose/hexose ratio were based only on three generalist species. We also found no statistically significant differences between generalists and specialists in nectar concentration, but the first orchid group secreted nectar that was more than 6% less concentrated than the second one. Generalist orchids produce nectar with the lowest concentration among the pollinator groups distinguished in orchids (18.31% ± 10.75%), but, among specialists with the longest spurs, the nectar was a little bit more diluted (17.73% ± 4.84%). How can this similarity between contrasting groups on the generalization–specialization scale be explained? In the case of specialists, the best explanation was provided by Nilsson’s [67] and Ambruster’s [68]. These authors stated that orchids, which accumulate nectar in the longest spurs, are pollinated by only one species of sphingid moth, thus have a more specialized pollination system than these offering nectar in shorter spurs, accessible to many species of noctuid and sphingid moths. The results of our analyses confirm this finding: orchids with the longest spurs (>5 cm) are pollinated on average by 1.7 pollinator species, whereas for orchids with spurs in the remaining 2 categories, the number of pollinators is 2 times higher (3.3 and 3.7, respectively). The statistically significant correlation between spur length and pollinator numbers (r = −0.28, *p* < 0.05) indicates higher specialization in orchids with longer spurs. Generalists must dedicate nectar to a wide range of mainly unspecialized pollinators, thus should produce nectar suitable for their differentiated mouth apparatus and equally differentiated dietary needs. It seems that less concentrated nectar does not restrict availability for different pollinator groups. This is confirmed by studies on the generalist *Neottia ovata*, which is pollinated by about 50 insect species [29]. At the species level, the plants’ offer is rich; nectar includes a wide range of components with high variability among populations, which exist in different communities, so are thus connected with different insect assemblages. This indicates that this species did not evolve nectar traits that filter flower visitors; thus, they are not dedicated to a certain group of pollinators.

### 3.3. Nectar Presentation 

Plant–pollinator interactions are influenced not only by the nectar quantity and quality, but also by its accessibility. Nectar accumulated inside the corolla or the spur is available for specific, restricted groups of pollinators, whereas exposed nectar may be collected by a wide range of pollinators from different taxonomic, morphological, and ecological groups [26,69]. In flowers with concealed nectaries, the nectar is dominated by sucrose, whereas in open nectaries, it is dominated by hexoses [20,21,32,40,41,42,66]. Nectar in deep flowers is protected against evaporation, and nectar in open nectaries is more vulnerable to this phenomenon (thus becoming viscous more quickly) and robbery [21,26]. It is also important that hexose nectars have lower evaporation rates due to their higher osmolarity, which explains the high proportion of hexoses in shallow flowers [70]. Nectar concentration and sugar ratios in Orchidaceae are similar, irrespective of the method of nectar presentation. Krömer et al. [45] found no relationship between flower morphology and sugar composition in Bromeliaceae, suggesting that sugar composition is, rather, correlated with pollinators, than with nectar presentation. Despite the lack of differences in the nectar concentration and sugar ratios between spurred orchids and those with open nectaries, in both groups of orchids we found examples that do not match the general pattern. Among the orchids with open nectaries included in our analyses, seven species have sucrose-dominant nectar [27,71,72,73] and five species produce nectar dominated by glucose and fructose [27,29,42,74,75]. In 1 species (*Caladenia nobilis*), the sucrose/hexose ratio is equal to 1. Hexose-rich nectar, whose uptake is easier compared with sucrose nectar, may be an adaptation and advantage for attracting a wide range of nonspecialized pollinators.

Although the nectar of spurred orchids did not differ from those with open nectaries, spur length influenced nectar traits; in shorter spurs, nectar appeared to be more concentrated and had a lower sucrose/hexose ratio. The fructose/glucose ratio was higher in longer spurs. A correlation between flower tube length and sucrose content in nectar was found by Witt et al. [46] in 78 European species of Caryophylloideae; the high sucrose content was linked with long flower tubes. The authors emphasized that long spurs or tubes restrict access to the nectar, thus reduce the diversity of pollinators; consequently, a high level of sucrose might be indicative of specialized pollination systems. Vandelook et al. [39] found a relationship between tube length and nectar traits in Balsaminaceae, but highlighted that it was overlaid by the pollination mode.

The nectar spurs are considered a key innovation promoting diversification in flowering plants [76], and the spur size is known to be an important feature determining which insect can act as a pollinator; therefore, the importance of spur length has been documented in studies on pollinator-mediated selection [77,78,79]. In conclusion, selection may work not only on nectar concentration, but also on corolla structure [39].

### 3.4. Taxonomy of Nectar

One of the most important aspects considered in nectar studies is plant phylogeny. According to Nicolson and Thornburg [21], phylogenetic history appears to be the primary determinant of nectar chemistry, and pollinators have a secondary effect. Krömer et al. [45] suggested that the patterns of distribution of nectar features vary across different plant groups and even proposed their use as diagnostic features. Generally, nectar traits may be highly variable in the taxonomic scale. Both differences between closely related taxa and similarities between divergent taxa were noted. This is well documented in studies on the ecosystem level, which included species belonging to many taxa living in similar conditions [20,39,49,50,51,54]. Witt et al. [46], studying nectar in Caryophyllaceae, showed that, in species from the *Dianthus* and *Saponaria* genera, sucrose-dominant nectars occur, whereas in *Sileneae*, the nectar is hexose rich. Additionally, *Silene* species show a dichotomy between species with sucrose-dominant vs. hexose-dominant nectars. Vandelook et al. [39] noted the lack of phylogenetic signal in Balsaminaceae and provided strong evidence that the evolution of nectar characteristics is a result of an adaptation to pollinator preferences. An interesting and well-illustrated example of this aspect is the data reported by Galetto and Bernardello [50] and Chacoff et al. [51], who found that *Embothrium coccineum* pollinated by passerines in Chile produces hexose-dominant nectar, but, when pollinated by hummingbirds in Argentina, the nectar is sucrose dominant. Pamminger et al. [56] recorded significant differences in nectar sugar concentration between distinct genera of plants pollinated by bees. The lack of significant differences in the nectar concentration and sugar ratio between two subfamilies (Epidendroidae and Orchidioidae) as well as between distinct genera, and the dependence of these traits on pollinator groups suggest that pollinator type is a primary factor in shaping nectar properties in Orchidaceae. Nevertheless, some genera are located on different edges of the overall variance; the lowest sugar concentration was observed for the *Angraecum* genus, whereas the highest for the *Rodriguezia*, *Bonatea*, and *Brownleea* genera. Species from the *Angraecum* genus had, on average, longer spurs than those from the three remaining genera. This may explain the differences in the nectar concentration. Another explanation of these differences may be their geography. The *Angraecum* genus was studied in Madagascar and Reunion Island and the others in South America or Africa. Notably, some species from *Angraecum* that have an extremely long spur (11.6 vs. 264 mm) produce nectar of similar concentrations. The most probable cause of these differences in nectar concentration within *Angraecum* is the pollinator type, as species with the lowest concentration are pollinated by birds, whereas those with a higher concentration by moths. This confirms the suggestion of Krömer et al. [45] that nectar characteristics are predominantly determined by putative adaptations of nectar sugars to the preferences of the pollinators, rather than by phylogenetic relationships. Opposite results were reported by Neubig et al. [54], who found that the highly variable sucrose concentration in Sobralieae is not related to pollinator type. Galetto et al. [27] found differences between two species from the *Habenaria genus* (*H. hieronymi* and *H. gourlieana*). Despite both being pollinated by moths, the former has a nectar concentration of 50.9% with 16.5% sucrose, whereas the latter has a concentration of 14.47% and almost 90% sucrose. This difference can be explained by the spur in *H. gourlieana*, which is 10 times longer than that in *H. hieronymi*. Perret et al. [58] suggested that plant–pollinator relationships rely on flower display rather than on nectar characteristics. Our data are too scarce to definitely state whether phylogenetic signals are important in Orchidaceae. It seems that it depends on the taxonomic level. To test whether phylogenetic constraints act on the nectar chemistry of orchids, studies on a wider range of species are needed.

## 4. Materials and Methods

### 4.1. Data Collection and Selection

To identify articles reporting data on nectar in Orchidaceae, we searched Google Scholar and Web of Science using the following keywords: “nectar orchid(s)” and “nectar Orchidaceae”. This searching method revealed only a few articles on the topic, because studies focusing on nectar in orchids are rare, to date. Therefore, in the next step, we precisely scanned the references from these publications and chose appropriate articles. In the majority of the papers, nectar analyses were only a small part of the studies on pollination biology or reproductive strategies in orchids. Therefore, we further searched scientific databases with the following keywords: “nectar pollination biology/reproductive strategy” and “orchid/Orchidaceae”. The returned papers (the ones cited in the text of the present article and [80,81,82,83,84,85,86,87,88,89,90,91,92,93,94,95,96,97,98,99,100,101,102,103,104,105,106,107,108,109,110,111,112,113,114,115,116,117,118,119,120,121,122,123,124,125,126]) were attentively read and used in the meta-analysis. Our original intent was to analyze nectar chemistry in orchids in a wider range, including detailed sugar and amino acid (AA) composition. Unfortunately, data about AAs in nectar of orchid flowers were only found in few publications. Therefore, our review was limited to sugars in floral nectar. Similarly, few datasets were obtained with detailed sugar composition. In effect, due to the features of the available data, we were only able to analyze the sugar concentration in the nectar of 106 orchid species from a wider context. In the cases in which sugar concentration was measured in small populations, we averaged the data from these populations. For some species, information about sucrose, fructose, and glucose concentration or their amount were available (N = 43). We used these data to analyze sucrose/hexose ratios (R1) following Baker and Baker [30], who distinguished four nectar categories: sucrose-dominant (R1 > 0.999), sucrose-rich (R1 = 0.999–0.5), hexose-rich (R1 = 0.499–0.1), and hexose-dominant (R1 < 0.1) categories. Additionally, we calculated the fructose/glucose ratios (R2).

Sugar concentrations and ratios between sugars were analyzed in the following contexts: geographical (continents and climatic zones), life forms (epiphytic or terrestrial), type of pollinators (we used only the data that reported the presence of pollinaria on animals bodies; thus, we report true pollinators in contrast with other studies, in which visiting animals were included), the level of specialization (specialist or generalist), and the method of nectar presentation (spur or open nectary). The level of specialization was defined after authors of each particular publication. Because the majority of the analyzed species accumulate nectar in spurs, we tested the influence of spur length on nectar concentration. We distinguished three spur categories: short (≤1 cm), medium (>1 and ≤5 cm), and long (> 5 cm).

### 4.2. Statistical Analyses

All statistical analyses were conducted in R. 4.0.3 (R Core Team). To test which factors explain sugar concentration in orchids, we built four sets of linear models. The logarithm of the response variable was used in the models to fit assumptions of normal distribution. The first model included only general habitat variables: climatic zone and life form. The second model included variables linked to the pollination mode: pollinator type, binary variable of pollinator match (specialist or generalist), as well as the spur length and nectar presentation (category of the spur length or open nectary). The third model covered all of the above-mentioned variables, and the fourth model covered only the climatic zone, pollinator type, and spur length. The most supported model was chosen according to the Akaike information criterion (AIC). Additionally, Bartlett’s test was conducted to check if the variance of nectar concentration (logarithm) differed between pollinator types (excluding beetles, which were represented by a single study in our meta-analysis). To check if the spur length affects the nectar concentration, an analysis of variance (ANOVA) was applied to the logarithm of nectar concentration. Next, Tukey’s post hoc test was applied to find out which pairs of spur length categories differ significantly.

The R1 and R2 ratios did not fit normal distribution; therefore, both were tested with a nonparametric test (Kruskal–Wallis) to check if sugar ratios are dependent on climatic zone, pollinator type, life form, and spur type. When significant differences were detected, Dunn’s multiple comparison test was applied to reveal the characteristics of the group of species that differentiated them from the others.

To check if the variance of sugar concentration differs between the studied genera of Orchidaceae, only the ones with data for at least 3 species per genus were used. Twelve genera fit this threshold and the logarithm of their sugar concentration variance was compared using Bartlett’s test. Additionally, the variance in two of the most numerous genera, *Satyrium* and *Habenaria*, was compared using Mood’s test. The variance in the R1 and R2 ratios and in the share of sucrose was tested between the different genera with the Fligner–Killeen test. Finally, the variance between Epidendroideae and Orchidoideae subfamilies was also tested with Mood’s test.

## 5. Conclusions

The knowledge of nectar traits in orchids and pollinators’ preferences of its properties agrees with one of the main questions in evolutionary biology. It highlights the importance of understanding the mechanisms of plant–pollinator interactions. The most important results of our analyses revealed that: 1. nectar properties in orchids show wide variability, similarly to other traits of this plant family; 2. nectar traits evolved in close association with pollinator types, thus are primarily shaped by pollinators; 3. although we found some differences in the nectar traits between orchids from distinct continents and climatic zones, it seems that they are derivatives of pollinator types existing in a given area and absent in another, as shown, for example, in the case of the hummingbirds.

The scarcity of data about nectar composition in orchids limits our answering of many interesting and important questions from evolutionary and ecological viewpoints. For example, the available data is predominantly focused on specialists; thus, we cannot accurately reach conclusions about nectar in generalists. Moreover, the limited data from some continents (e.g., Asia, Australia, Europe, and North America) do not enable a discussion about the geography of nectar. Because only a few papers reported amino acid compounds in orchid nectar, it is impossible to discuss their role in pollinators’ preferences. The knowledge of AAs in nectar is important as, through them, plants possess the potential to manipulate pollinators. The next questions to consider are how nectar chemistry is involved in the adaptation to a specific pollinator spectrum and how phylogenetic constraints act on nectar chemistry. To answer these questions, data on a wider range of orchid species are needed. Our review is the first step in compiling knowledge on nectar properties in orchids and we hope that it will be helpful for future studies on the nectar of this family.

## Figures and Tables

**Table 1:** Results of the most-supported linear model explaining the logarithm of sugar concentration in orchids.

	Log (Sugar Concentration)		
Predictors	Estimates	CI	*p*
(Intercept)	1.77	0.77–2.76	0.001
Continent [Asia]	1.16	0.30–2.02	0.009
Continent [Europe]	0.68	−0.29–1.66	0.166
Continent [N. America]	0.74	−0.29–1.78	0.158
Continent [S. America]	0.33	0.08–0.58	0.012
Climate zone [southern temperate]	1.06	0.13–1.99	0.027
Climate zone [tropics]	0.69	−0.19–1.57	0.124
Pollinators [bees]	0.62	0.25–0.99	0.001
Pollinators [beetles]	−2.41	−3.62–−1.20	<0.001
Pollinators [butterflies]	0.45	−0.02–0.92	0.060
Pollinators [flies]	0.45	−0.04–0.94	0.073
Pollinators [hummingbirds]	0.13	−0.25–0.50	0.500
Pollinators [moths]	0.27	−0.09–0.64	0.137
Spur category [no spur, open nectaries]	0.05	−0.23–0.33	0.744
Spur category [below 1 cm]	0.07	−0.19–0.34	0.576
Spur category [over 5 cm]	−0.09	−0.37–0.20	0.553
Observations	106		
R^2^/R^2^ adjusted	0.491/0.406		

## Data Availability

The key data used for the meta-analysis is available in the online Supplementary Materials in Table S1.

## Supplementary Materials

The following are available online at https://www.mdpi.com/article/10.3390/plants10112315/s1: Table S1. Properties of analyzed orchid traits, including nectar characteristics.; Table S2. Sugar ratios in Orchidaceae in comparison with Baker and Baker (1983a) according to pollinator type. Data include the number of species studied with percentages in parentheses within the pollinator type.; Table S3. Composition and performance of linear models explaining the logarithm of sugar concentration in flowers of 106 orchid species.; Table S4. Results of the Kruskal–Wallis tests comparing sugar ratios in the nectar of orchid species depending on their geographical location, habitat, and pollination mode.; Figure S1. Variance in sugar concentration in flowers of different genera in the Orchidaceae family.; Figure S2. Spur length of different pollinator groups of 105 orchids included in our meta-analysis of nectar composition of the Orchidaceae (the single species pollinated by beetles was omitted).
