# Supplementary material for: Floral Nectar Chemistry in Orchids: A Short Review and Meta-Analysis
Source: Plants (Basel). 2021 Oct 27;10(11):2315. doi: 10.3390/plants10112315 (PMC8620889; doi:10.3390/plants10112315)
Supplement: Supplementary file 1 [file plants-10-02315-s001.zip › plants-1425065-supplementary.pdf]

Table S1. Properties of analyzed orchid traits, including nectar characteristics. Life form: T- terrestrial, E - epiphitic, El - epilithic; pollinators: B - bees, M - moths, H - birds, Bu - butterflies, F - flies, W- wasps, Bt - beetles, D - various; pollinator specialization: S - specialist, G - generalist; nectar presentation: S - in spur, O - in open nectaries; spur category: S - short, M - medium, L - long.

Species	Subfamily	Continent	Life form	Pollinators	Specialist/ generalist	Nectar presentation	Spur length (mm)	Spur category	Sugar concentration (%)	S:G+F	F:G	Source
Acrolophia cochlearis	Epidendroideae	Africa	T	B	S	S	3	S	90	2.33	0.88	Peter &Johnson 2009
A. micrantha	Epidendroideae	Africa	T	B	S	S	3	S	71	-	-	Peter &Johnson 2009
Aeranthes arachnitis	Epidendroideae	Africa	E	M	S	S	-	M	14.5	-	-	Roberts 2007
Aerangis brachycarpa	Epidendroideae	Africa	E	M	S	S	159	L	22.5	-	-	Martins & Johnson 2007
A. confusa	Epidendroideae	Africa	El	M	S	S	45	M	23	-	-	Martins & Johnson 2007
A. ellisii	Epidendroideae	Africa	E.	M	S	S	158	L	16.2	-	-	Nilsson & Rabakonandrianina
A. thomsonii	Epidendroideae	Africa	E	M	S	S	135	L	14	-	-	1988
A. verdickii	Epidendroideae	Africa	E	M	S	S	150	L	16.35	-	-	Martins & Johnson 2007 Koopowitz & Marchant 1998
Rangaeris amaniensis	Epidendroideae	Africa	E	M	S	S	156	L	20.5	-	-	Martins & Johnson 2007
Angraecum arachnites	Epidendroideae	Africa	E	M	S	S	100	L	13.3	-	-	Nilsson et al. 1985; Johnson & Raguso 2016
A. striatum	Epidendroideae	Africa	E	H	S	S	11.6	M	9.7	-	-	Micheneau et al. 2006
A. bracteosum	Epidendroideae	Africa	E	H	S	S	8	S	9.6	-	-	Micheneau et al. 2008
A. sesquipedale	Epidendroideae	Africa	E	M	S	S	333	L	16.5	-	-	Wasserthal 1997
A. sororium	Epidendroideae	Africa	El	M	S	S	264	M	10.3	-	-	Wasserthal 1997
Beadlea dufrae		S. America	T	B	S	O	-	-	60.45	18.7	0.72	Galetto et al. 1997
Bonatea cassidea	Orchidoideae	Africa	T	Bu	S	S	20.8	M	27.9	67.50	3.29	Balducci et al. 2019
B. polypodantha	Orchidoideae	Africa	T	M	S	S	43.9	M	20	4.34	1.14	Balducci et al. 2020
B. speciosa	Orchidoideae	Africa	T	M	S	S	50	M	27.3	-	-	Johnson & Liltved 1997; Ponsie et al. 2007
B. macroceras	Orchidoideae	Africa	T	F	S	S	36.2	M	30.5	-	-	Larsen et al. 2008
B. parviflora	Orchidoideae	Africa	T	B	S	S	3.7	S	33.43	-	-	Larsen et al. 2008
Caladenia arenaria	Orchidoideae	Australia	T	W	S	O	-	-	-	19.00	1	Reiter et al. 2019b
C. colorata	Orchidoideae	Australia	T	W	S	O	-	-	-	19.00	1	Reiter et al. 2018
C. concolor	Orchidoideae	Australia	T	W	S	O	-	-	-	-	-	Reiter et al. 2019b
C. nobilis	Orchidoideae	Australia	T	W	S	O	-	-	-	1.00	1.6	Phillips et al. 2019
C. paludosa	Orchidoideae	Australia	T	W	S	O	-	-	-	2.37	1.02	Phillips et al. 2019
C. versicolor	Orchidoideae	Australia	T	B	S	O	-	-	-	19.00	1	Reiter et al. 2019a
Cleisostoma yersinii	Epidendroideae	Asia	E	B	S	S	10	S	74.51	20.89	0.84	Ponert et al. 2016
Comparettia coccinea	Epidendroideae	S. America	E	Bu	S	S	18	M	22.5	-	-	Pansarin et al. 2015

C. falcata	Epidendroideae	S. America	E	H	S	S	13.1	M	14	-	-	Rodríguez-Robles et al. 1992
Cynorkis uniflora	Orchidoideae	Africa	T	M	S	S	-	-	18.4	-	-	Nilsson et al. 1992
Cyrtorchis arcuata	Epidendroideae	Africa	E	M	S	S	33	M	16.65	-	-	Luyt 2002
Diaphananthe caffra	Epidendroideae	Africa	E	M	S	S	14	M	14.86	4.26	1.1	Luyt 2002
D. millarii	Epidendroideae	Africa	E	M	S	S	18.1	M	13.42	-	-	Luyt 2002
Disa chrysostachya	Orchidoideae	Africa	T	H	S	S	7.7	S	9.9	-	-	Johnson & Brown 2004
D. cooperi	Orchidoideae	Africa	T	M	S	S	42	M	34.9	0.89	1.3	Johnson 1995; Johnson 2006
D. satyriopsis	Orchidoideae	Africa	T	H	S	S	10.9	M	14.7	-	-	Johnson & Brown 2004
D. scullyi	Orchidoideae	Africa	T	F	S	S	42	M	30.3	3.0	0.79	Johnson 2006
Elleanthus aurantiacus	Epidendroideae	S. America	T	H	S	O	-	-	23.5	-	-	Neubig et al. 2015
E. brasiliensis	Epidendroideae	S. America	E	H	S	O	-	-	21.11	27.90	1.37	Nunes et al. 2013
E. caravata	Epidendroideae	S. America	E	H	S	O	-	-	26	-	-	Neubig et al. 2015
E. cynarocephalus	Epidendroideae	S. America	E	H	S	O	-	-	13	-	-	Neubig et al. 2015
E. sodiroi	Epidendroideae	S. America	T	H	S	O	-	-	16	-	-	Neubig et al. 2015
Epipactis atropurpurea	Epidendroideae	Europe	T	D	G	O	-	-	20	0.27	0.8	Pais & Neves 1980
E. atropurpurea	Epidendroideae	Europe	T	D	G	O	-	-	32	-	-	Pais et al. 1986
Eulophia alta	Epidendroideae	S. America	T	B	S	O	-	-	-	0.80	1.00	Jürgens et al. 2009
Gymnadenia conopsea	Orchidoideae	Europe	T	Bu	S	S	18	M	-	4.20	0.76	Gijbels et al. 2014
G. odoratissima	Orchidoideae	Europe	T	F	S	S	4.55	S	15.64	-	-	Sun et al. 2014
Habenaria aitchisonii	Orchidoideae	Asia	T	M	S	S	9	S	27.11	-	-	Xiong et al. 2019
H. davidii	Orchidoideae	Asia	T	M	S	S	63.98	L	24.5	-	-	Zang et al. 2017
H. epipactidea	Orchidoideae	Africa	T	M	S	S	29.5	M	27.9	-	-	Peter et al. 2009
H. fordii	Orchidoideae	Asia	T	M	S	S	65.31	L	16.47	-	-	Zang et al. 2017
H. glaucifolia	Orchidoideae	Asia	T	M	S	S	25.22	M	30.55	-	-	Xiong et al. 2015
H. gourlieana	Orchidoideae	S. America	T	M	S	S	133.9	L	14.47	7.94	0.75	Galetto et al. 1997, Singer &Cocucci 1997
H. hieronymi	Orchidoideae	S. America	T	M	S	S	13.2	M	50.9	0.20	2.05	Galetto et al. 1997; Singer & Cocucci 1997
H. johannensis	Orchidoideae	S. America	T	M	S	S	13.8	M	14.6	-	-	Amorim et al. 2014
H. johannensis	Orchidoideae	S. America	T	M	S	S	130.9	L	24.46	-	-	Pedron et al. 2012
H. limprichtii	Orchidoideae	Asia	T	M	S	S	19.22	L	26.2	-	-	Tao et al. 2018
H. macronectar	Orchidoideae	S. America	T	M	S	S	66.6	L	18.12	-	-	Pedron et al. 2012
H. megapotamensis	Orchidoideae	S. America	T	M	S	S	75.1	L	19.04	-	-	Pedron et al. 2012
H. montevidensis	Orchidoideae	S. America	T	Bu	S	S	16.2	M	26.2	-	-	Pedron et al. 2012
H. paulistana	Orchidoideae	S. America	T	M	S	S	12.9	M	19.2	-	-	Amorim et al. 2014
H. pleiophylla	Orchidoideae	S. America	T	M	S	S	22.3	M	40	-	-	Singer et al. 2007
H. ornithoides	Orchidoideae	S. America	T	M	S	S	40	M	-	27.09	2.16	Gottsberger et al. 1984
H. rhodocheila	Orchidoideae	Asia	T	Bu	S	S	41.26	M	20.89	-	-	Chen et al. 2021
Jumellea stenopylla	Epidendroideae	Africa	E	M	S	S	137.9	L	10.7	-	-	Micheneau et al. 2008
Limodorum abortivum	Orchidoideae	Europe	T	B	G	S	15	M	16.9	-	-	Pais et al. 1986; Vakhremeeva
Luisia teres	Epidendroideae	Asia	E	Bt	S	O	-	-	3.5	-	-	Arakaki et al. 2016
Maxillaria anceps	Epidendroideae	S. America	E	B	S	O	-	-	66.5	70.86	0.44	Davies et al. 2005

Mystacidium brayboniae	Epidendroideae	Africa	E	M	S	S	19.4	M	11.42	46.00	-	Luyt 2002
M. capense	Epidendroideae	Africa	E	M	S	S	39.1	M	15.96	7.30	2.45	Luyt 2002
M. flanaganii	Epidendroideae	Africa	E	M	S	S	19.6	M	23.6	1.38	1.21	Luyt 2002
M. gracile	Epidendroideae	Africa	E	M	S	S	25.6	M	18.26	2.45	0.96	Luyt 2002
M. pusillum	Epidendroideae	Africa	E	M	S	S	20.4	M	21.46	1.27	0.83	Luyt 2002
M. pusillum	Epidendroideae	Africa	E	M	S	S	20.4	M	24.2	-	-	Peter & Venter 2016
M. tanganyikense	Epidendroideae	Africa	E	M	S	S	16.8	M	-	4	1.5	Luyt 2002
M. venosum	Epidendroideae	Africa	E	M	S	S	47	M	16	6.70	3.33	Luyt & Johnson 2001; Luyt 2002
Neottia ovata	Epidendroideae	Europe	T	D	G	O	-	-	3.39	0.22	0.88	Brzosko et al. 2021
Oececoclades maculata	Epidendroideae	S. America	T	Bu	S	S	5	S	33.73	-	-	Aguiar & Pansarin; Gonzales-Dias, Ackerman 1988
Platanthera chlorantha	Orchidoideae	Europe	T	M	S	S	30	M	19.37	-	-	Stpiczyńska & Pielecki 2002
P. bifolia	Orchidoideae	Europe	T	M	S	S	27.9	M	12.07	0.32	0.67	Brzosko & Bajguz 2019
P. chlorantha	Orchidoideae	Europe	T	M	S	S	32.37	M	15.78	0.09	0.61	Brzosko & Bajguz 2019
P. ciliaris	Orchidoideae	N. America	T	Bu	S	S	23.7	M	21	-	-	Robertson & Wyatt
P. praeclara	Orchidoideae	N. America	T	M	S	S	44.5	M	24.7	-	-	Westwood et al. 2011
P. stricta	Orchidoideae	N. America	T	D	S	S	3.97	S	8	-	-	Patt et al. 1989
Pelexia bonariensis	Orchidoideae	S. America	T	B	S	O	-	-	39.55	1.70	0.57	Galetto et al. 1997
Pteroglossa glazioviana	Orchidoideae	S. America	T	Bu	S	S	13	M	26.75	-	-	Pansarin & Ferreira 2015
P. roseoalba	Orchidoideae	S. America	T	Bu	S	S	31	M	27.98	-	-	Pansarin & Ferreira 2015
Rhynchostylis retusa	Epidendroideae	Asia	E	B	S	S	5	S	45.7	-	-	Buragohain et al. 2015
Rodriguezia bahiensis	Epidendroideae	S. America	E	B	G	O	-	-	16.57	-	-	Carvalho & Machado 2006
R. granadensis	Epidendroideae	S. America	E	B	S	S	26.5	M	33	-	-	Ospina-Calderón et al. 2015
R. lanceolata	Epidendroideae	S. America	E	H	S	S	3	S	36	-	-	Pansarin et al. 2018
Satyrium carneum	Orchidoideae	Africa	E	H	S	S	23.4	M	31.2	-	-	Johnson 1996
S. coriifolium	Orchidoideae	Africa	T	H	S	S	12.3	M	23.6	-	-	Johnson 1996; Ellis and Johnson 1999
S. hallackii ssp. hallackii	Orchidoideae	Africa	T	B	S	S	13.6	M	23.1	1.63	1	Johnson 1997
S. hallackii ssp. ocellatum	Orchidoideae	Africa	T	D	S	S	32.8	M	18.9	2.70	1.08	Johnson 1997
S. princeps	Orchidoideae	Africa	E	H	S	S	21.9	M	28.6	-	-	Johnson 1996
S. rhodanthum	Orchidoideae	Africa	T	H	S	S	31.89	M	24.8	-	-	van der Niet et al. 2015
S. crassicaule	Epidendroideae	Africa	T	M	S	S	16	M	23.3	2.23	1.07	Johnson et al. 2011
S. cristatum var. cristatum	Epidendroideae	Africa	T	B	S	S	10	S	27.5	1.63	0.8	Johnson et al. 2011
S. cristatum var.longilabiatum	Epidendroideae	Africa	T	B	S	S	10	S	35.1	-	-	Johnson et al. 2011
S. longicauda	Epidendroideae	Africa	T	M	S	S	40	M	31.2	4.00	1	Johnson et al. 2011
S. macrophyllum	Epidendroideae	Africa	T	F	S	S	33	M	27.9	100.00	-	Johnson et al. 2011
S. monadenum	Epidendroideae	Africa	T	H	S	S	18	M	16.4	-	-	Johnson et al. 2011
S. neglectum	Epidendroideae	Africa	T	D	G	S	16	M	31.3	1.70	0.95	Johnson et al. 2011
S. parviflorum	Epidendroideae	Africa	T	M	S	S	12	M	15.7	1.94	1.27	Johnson et al. 2011
S. sceptrum	Epidendroideae	Africa	T	H	S	S	37.9	M	18.7	7.33	1	Johnson et al. 2011
S. sphaerocarpum	Epidendroideae	Africa	T	B	S	S	13	M	24.6	100.00	-	Johnson et al. 2011
S. trinerve	Epidendroideae	Africa	T	D	S	S	5.96	S	12.4	-	-	Johnson et al. 2011
Sobralia bouchei	Epidendroideae	S. America	E	B	S	O	-	-	20	-	-	Neubig et al. 2015
S. callosa	Epidendroideae	S. America	E	H	S	O	-	-	15.75	-	-	Neubig et al. 2015
S. macrophylla	Epidendroideae	S. America	E	B	S	O	-	-	20	-	-	Neubig et al. 2015

S. rosea	Epidendroideae	S. America	T	B	S	O	-	-	12.25	-	-	Neubig et al. 2015
Stenorrhynchos orchioides	Orchidoideae	S. America	T	H	S	O	-	-	43.9	0.5	0.8	Galetto et al. 1997
Schizochilus flexuosus	Orchidoideae	Africa	T	B	S	S	4.73	S	30.25	-	-	Van der Niet et al. 2010
S. zeyheri	Orchidoideae	Africa	T	F	S	S	3.36	S	22.1	-	-	Van der Niet et al. 2010

Table S2. Sugar ratios in Orchidaceae in comparison with Baker and Baker (1983a) according to pollinator type. Data include the number of species studied with percentages in parentheses within the pollinator type.

**Nectar sugar ratio**	**Source**	**Bird-pollinated species**	**Bee-pollinated species**	**Moth-pollinated**	**Butterfly-pollinated species**	**Fly-pollinated species**	**Wasp-pollinated species**	**Generalist**
Suchrose dominant	Present work	2 (66.6)	9 (100)	14 (77.8)	2 (100)	2 (100)	2 (66.6)	2 (50)
	Baker & Baker	77(55.0)	83 (17.8)	47 (45.2)	29 (38.7)			
Sucrose rich	Present work	0	0	1 (5.6)	0	0	1 (33.3)	0
	Baker & Baker	45(32.1)	77 (16.5)	30 (28.8)	24 (32.0)			
Hexose rich	Present work	1 (33.3)	0	2 (11.1)	0	0	0	2 (50)
	Baker & Baker	18 (12.8)	178 (38.2)	22 (21.1	17 (22.6)			
Hexose dominant	Present work	0	0	1 (5.6)	0	0	0	0
	Baker & Baker	0(0)	128 (27.5)	5 (4.8)	5 (6.6)			

Table S3. Composition and performance of linear models explaining the logarithm of sugar concentration in flowers of 106 orchid species

#	Variables included	df	AIC	ΔAIC
1	Continent + climatic zone + habitat	11	152.00	28.66

2	Pollinator type + pollinator match + spur type	12	136.30	12.96
3	Continent + climatic zone + habitat + pollinator type + pollinator match + spur type	21	126.84	3.50
4	Continent + climatic zone + pollinator type + spur type	17	123.34	0

Table S4. Results of the Kruskal–Wallis tests comparing sugar ratios in the nectar of orchid species depending on their geographic location, habitat, and pollination mode.

Factor	χ^2	df	p
Saccharose to hexoses ratio			
Continent	8.07	3	0.044
Climatic zone	10.99	2	0.0041
Habitat	2.58	1	0.11
Pollinator type	6.83	5	0.23
Pollinator match	0.73	1	0.39
Spur type	0.75	3	0.86
Fructose to glucose ratio			
Continent	8.34	3	0.039
Climatic zone	4.13	2	0.13
Habitat	1.23	1	0.27
Pollinator type	9.59	5	0.088
Pollinator match	2.06	1	0.16
Spur type	7.68	3	0.053

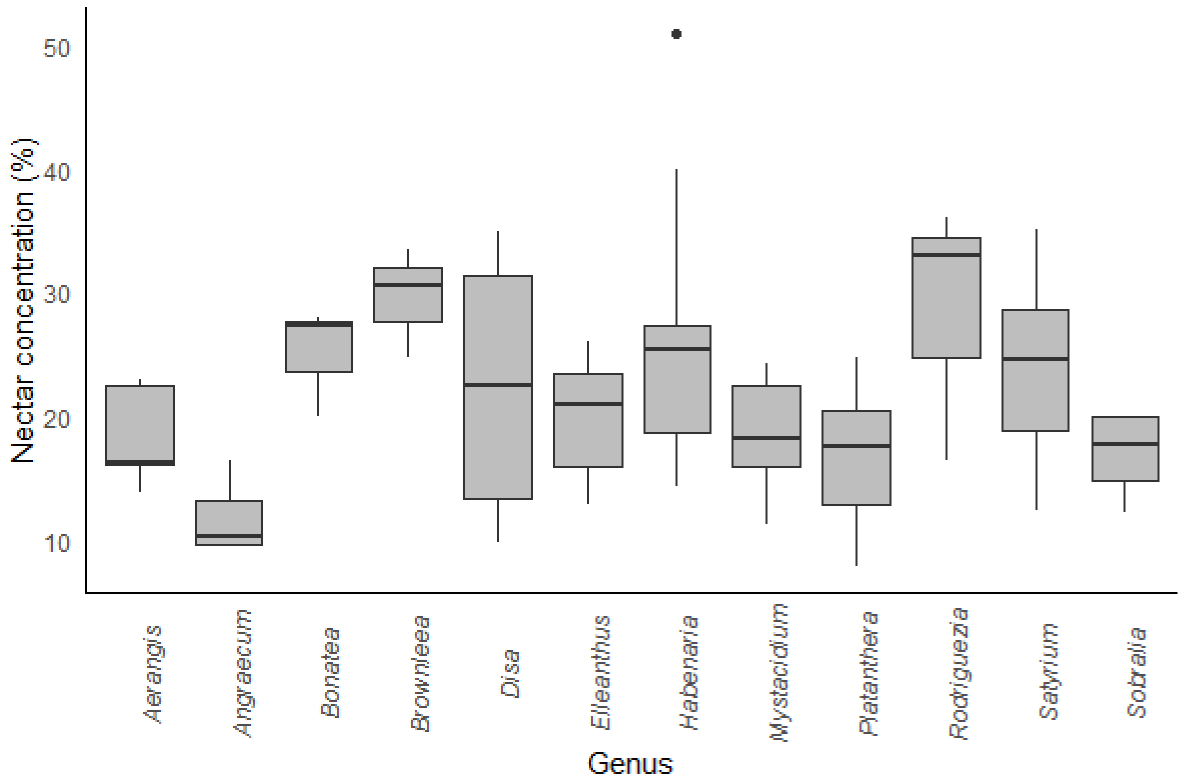

Figure S1. Variance in sugar concentration in flowers of different genera in Orchidaceae family

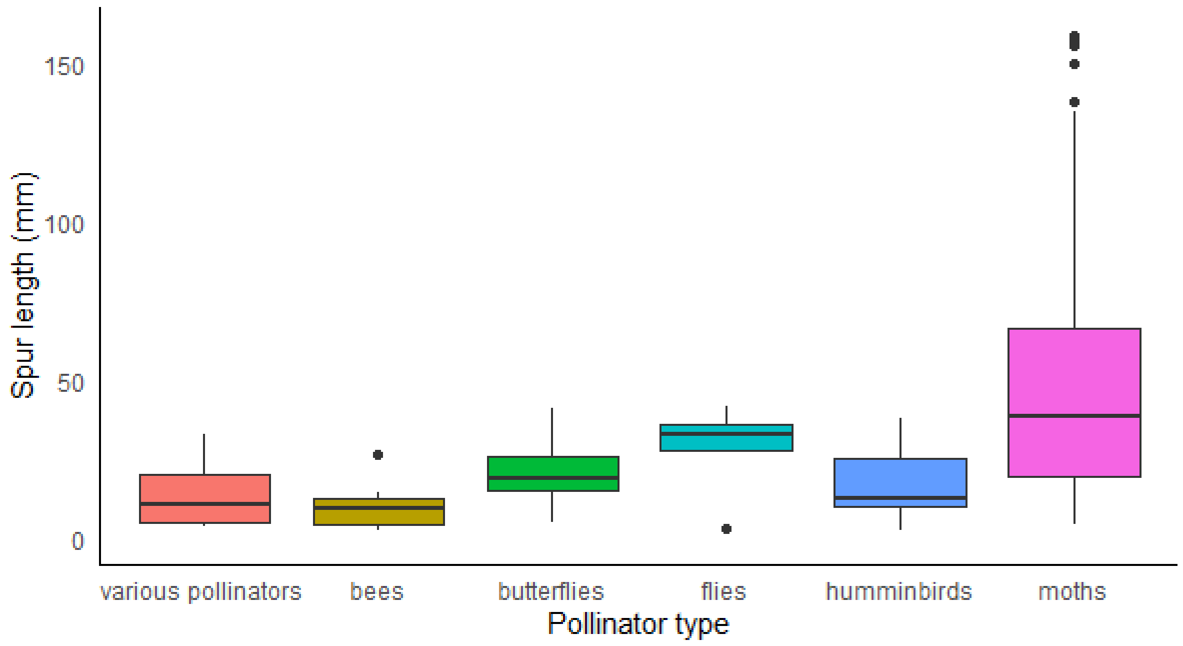

Figure S2. Spur length of different pollinator groups of 105 orchids included in our meta-analysis of nectar composition of the Orchidaceae (the single species pollinated by beetles was omitted).
